# 

**Published:** 1883-02

**Authors:** 


### CHICAGO MEDICAL JOURNAL & EXAMINER.

CHICAGO MEDICAL PRESS ASSOCIATION.

AUG. 2D

1875.

CHICAGO:
*PUBLISHED BY THE CHICAGO MEDICAL PRESS ASSOCIATION*
188 CLARK STREET.

**Read the Notice of this Journal among Advertisements.**

